# Certified Examination Assistants in the Age of Telemedicine: A Blueprint Through Neurology

**DOI:** 10.2196/28335

**Published:** 2021-10-06

**Authors:** Ilya Bragin, Dylan T Cohen

**Affiliations:** 1 St. Luke's University Health Network Bethlehem, PA United States; 2 Lewis Katz School of Medicine at Temple University Philadelphia, PA United States; 3 SUNY Downstate Medical Center Brooklyn, NY United States

**Keywords:** telemedicine, physical examination, neurological exam, telemedicine assistants, telemedicine implementation, telemedicine certification, telemedicine jobs, telemedicine education, telehealth, teleneurology

## Abstract

The optimal approach to a clinical physical examination via telemedicine is still being explored. The medical community has no standardized or widely followed criteria for telemedicine examinations, so a broad spectrum of approaches is used. Unfortunately, the need for telemedicine is outpacing physical examination validation research. Given that certain specialties have been using telemedicine longer than others, lessons from those specialties might aid in developing standardized protocols for telemedicine. Neurology has been at the forefront of telemedicine use, initially through stroke care and later in multiple subspecialties. We present a framework for optimizing the history taking and physical examination process via telemedicine based on our experience in neurology. This mainly includes remotely examining a patient unassisted or with an untrained assistant present on the patient side of the connection. We also discuss the need for trained, certified assistants to assist the off-site physician in history taking and physical examination. These certified assistants would be allied health professionals who perform high-quality cued patient examinations under direct physician supervision with no responsibility to diagnose or treat. This contrasts with the approach seen in advanced practice providers such as physician assistants and nurse practitioners who undergo years of training to diagnose and treat patients under supervision. This training process would serve as a stepping stone for the development of dedicated certification programs for neurology and other medical specialties; however, assessments of practical training, costs, implementation, and longitudinal quality are warranted.

## Commentary

The criterion standard for physical examination is in-person examinations; however, the quality of in-person examinations varies considerably based on the examiner’s education and experience, even for physicians and advanced practice providers (APPs) of different specialties. Widespread telemedicine implementation has facilitated access to medical care worldwide but not without particular challenges. For example, history taking is easily translatable to a virtual setting, but physical examination can be problematic. This is especially true in specialties with extensive physical examinations using telehealth models for some time, such as neurology [1]. We have had years of experience using in-person examinations in conjunction with laboratory data, neurophysiological studies, and imaging data to provide high-value neurological care.

Currently, a wide assortment of telehealth equipment exists to aid the remote health care provider. Generalized digital cameras can function variably as otoscopes, ophthalmoscopes, and dermatoscopes, and can be used to perform specialized examinations. Some telemedicine units are equipped with electronic stethoscopes to transmit data to be interpreted by the remote examiner [2]. These tools are a means of replicating the in-person clinical environment and decrease barriers to a comprehensive examination. Although technical equipment may vary, minimum requirements include high-speed internet, a good quality camera with zooming capability, and the space to adequately position the camera at appropriate distances from the patient to be able to see the body as a whole and to zoom in on parts such as pupils. However, although the technical equipment can be reproduced on a mass scale and become readily available, replacing the skills and intuition of a clinical examiner gained through years of training and in-person experience cannot be so easily replaced. Physicians are not the only providers assessing patients; nursing staff (including nurse practitioners, registered nurses, licensed practical nurses, and nursing assistants) tend to engage in more than 2.5 times the amount of direct bedside care than other medical staff (including physicians, physician assistants, and medical students) [3]. The presence of an additional health care provider represents an untapped valuable resource for telemedicine. These providers can exist in a variety of settings, including inpatient hospitals, outpatient clinics, rehabilitation facilities, long-term care facilities, and the patients’ homes. Though resources vary depending on location, providing a general framework to performing a virtual examination can help create consistency across the board, and certified assistants can be used in any of these settings.

Certification programs geared explicitly toward physical examination skills are varied and still in the early adoption phase. For example, Thomas Jefferson University offers an online 5-week program to become a certified “Telehealth Facilitator” that imparts the skills necessary to manage the interface between the provider and patient, and to address any arising technological problems during the encounter [4]. The National School of Applied Telehealth has an online 3- to 5-hour course that provides telemedicine knowledge basics to allow students to properly present virtual patients to clinicians as a “Certified Telemedicine Clinical Presenter.” However, this program does not teach physical examination skills and assumes the student is already proficient in this area [5]. An online course is not likely to equip a student with the skills and experience needed to perform a physical examination well. One study at a university hospital in China enrolled 72 fourth-year medical students in a formal 17-week training course for physical examinations and found an error rate of 1.097 errors per student per body system examined [6], which highlights the need for ongoing student-patient interactions to gain proficiency in performing physical examinations. There are other programs designed for APPs to specialize in various fields like neurology, but these programs focus on complete management and treatment and are time-consuming, expensive, and well beyond the scope required to assist a clinician for a telehealth encounter remotely.

Given that neurology relies heavily on detailed history taking and precise physical examination to localize lesions, its success in the telemedicine environment can serve as a framework for telehealth implementation in other fields. Initial applications of teleneurology included acute stroke care with validation of the National Institute of Health Stroke Scale performed remotely. General neurological examination via teleneurology research is ongoing to assess the reproducibility and reliability of remote neurological examination compared to in-person examinations. The adoption of telemedicine technology is outpacing the literature regarding accuracy and reliability, and although data are emerging, pragmatic models for implementation and use are needed now. The legal implications of relying on remote assistants are unclear. Causes of telemedicine litigation can typically be related to issues with informed consent, breaking state or federal laws, data breaches, diagnostic errors, and lack of policies and protocols. Trained assistants can facilitate informed consent and help provide a higher quality examination leading to fewer diagnostic errors. This would be expected to diminish litigation and improve outcomes.

The optimal way to perform a neurological physical examination and take a patient’s history remotely has been a challenge since 1999 when it was first reported possible [7]. COVID-19 has created an urgent need for new attempts to educate the neurology community with instructional videos and implementation guides [8,9]. However, formal criteria for performing a neurological examination that uses a telehealth assistant have not been established to our knowledge. In a recent newsletter, The Joint Commission published important considerations in optimizing patient care via telehealth but left protocol structuring to local organizations and providers. One consideration mentions appropriately training staff and defining roles and responsibilities [10]. Additionally, following the outbreak of the COVID-19 pandemic, the American Medical Association released a telemedicine implementation guide highlighting the importance of familiarizing all essential health care team members with telehealth platforms. However, although the guide offered some practical generalizations, there were no specifications regarding assistance in a neurological examination [11].

The skill in performing and interpreting the neurological exam and the comfort level under various circumstances are arguably more important than whether the exam is in-person or remote. Additionally, the reliability of neurological exams will vary even among the best health care providers. Kappa statistics can measure test interrater reliability with higher scores indicating higher levels of agreement (ie, 0.01-0.20: slight agreement; 0.21-0.40: fair agreement; 0.41-0.60: moderate agreement; 0.61-0.80: substantial agreement; and 0.81-0.99: almost perfect agreement). For neurological examinations, kappa scores are better among observable signs than elicitable neurologic signs. In other words, parts of the examination with high reliability required the examiner to do less elicitation and observe [12]. One telemedicine study showed kappa statistics for assessing language, tongue movement, and finger-to-nose coordination were 0.82, 0.69, and 0.68, respectively [13]. However, maneuvers that required more skill on the examiner’s part resulted in lower scores: reflex scores in that same study ranged from 0.38 to 0.52, visual field assessments were 0.56, and pinprick tests were 0.56 [13].

The teleneurology paradigm involves many elements, and this paper provides a logistical framework for the patient history and physical examination elements of the encounter. Importantly, these aspects can vary substantially based upon individual patient characteristics, the availability of remote assistance, and the assistant’s training.

The acquisition of patient history should not be altered substantially due to the lack of in-person interaction. If a patient is cognitively intact, they may provide their history. If not, history may be obtained via a surrogate (eg, a family member or nursing staff), as would be the case during an in-person encounter. There is an opportunity to improve the history-taking process. During telemedicine visit preparation, the health care team can schedule coordinated group calls or video chats between the patient, family, and provider to optimize history taking. Geographically dispersed family members and caretakers can contribute to complex histories. This would add substantially to the patient history, as patients are often limited historians, and physicians must comb through the electronic medical records to piece together a story that is often documented asynchronously or even incorrectly [14]. Whether the latter is due to false patient recall or vague physician documentation, the matter is irrelevant—having key individuals sitting in on the same call may enable the acquisition of a single cohesive history.

Although the history gathering portion of the patient encounter is relatively straightforward, a teleneurology examination presents challenges. Nevertheless, all encounters (both assisted or unassisted) can still include the standard seven parts of a neurologic examination (ie, assessment of mental status, cranial nerves, motor system, sensory system, reflexes, cerebellar, and gait). Each subsection of the exam can be modified as needed based on the degree of assistance available (Table 1); however, a genuinely comprehensive neurological examination would require a well-trained assistant. Multimedia Appendix 1 [15] presents a detailed breakdown of the neurological examination elements.

Specially trained and certified examiners have an important role in helping to conduct specialty examinations in the telehealth setting. Although the certification process could potentially be streamlined for those who are already capable of performing adequately directed physical examinations (eg, physician assistants or nurse practitioners), full-scale physical examination–specific training and certification would be advantageous for other hospital and office staff and trainees, including nurses, hospital technicians (eg, those in neurophysiology and respiratory staff), medical trainees (eg, medical students and advanced practitioner students), and patient care assistants. This is important as the certified examiners would likely have other primary clinical responsibilities. Roles and titles must clearly be defined, as a lack of consistency in nomenclature across medical settings has led to false assumptions of tele-assistant capabilities when working with other health care staff [16]. Training would need to focus on multiple real patient physical examinations supervised by a board-certified specialty physician. Training must also focus on quality and reproducibility rather than the rote memorization and checklist-style grading often used by formal testing (eg, objective-structured clinical examinations). Subspecialty examination certifications can be considered. Importantly, unlike APPs trained over multiple years to treat patients under supervision, these certified health professionals would not be expected to offer assessment or provide treatment and function as a direct extension of the tele-examiner. This is a critical difference because training would focus solely on the examination and not the management aspect of the patient encounter, which would remain purposefully the exclusive responsibility of the telemedicine clinician.

**Table 1:** Neurological examination capabilities based on assistant availability and expertise.

Exam maneuver		TA^a^	UTA^b^	UA^c^	Comments
Mental status		✓	✓	✓	May be limited at times based on degree of cognitive impairment, regardless of etiology (eg, dementia, delirium, or static encephalopathy). Certain elements such as cortical sensory testing/diagnosis would require a trained assistant.
**Cranial nerves**					
	Olfactory	✓	✓	X	Given assistant has access to scent.
	Visual acuity	✓	✓	✓	Visual acuity mobile apps are readily available.
	Extraocular movements	✓	✓	✓	Saccades, smooth pursuit, convergence can be assessed by all three levels. Oculocephalic maneuvers would need TA.
	Pupillary response	✓	✓	✓	Direct pupillary reflex can be assessed with eye opening/closing. Indirect needs TA.
	Facial sensation	✓	✓	X	Gross sensation only with TA and UTA. Multimodal sensation only with TA.
	Taste	✓	X	X	N/A^d^
	Audition	✓	✓	X	Weber/Rinne testing needs TA.
	Vestibular	✓	X	X	N/A
	Articulation	✓	✓	✓	N/A
	Swallowing	✓	✓	✓	Via observing drinking and eating
	Trapezius and SCM^e^	✓	✓	✓	Cannot assess strength unassisted but can assess symmetry
	Tongue	✓	✓	✓	Strength only with TA
**Motor**					
	Observation	✓	✓	✓	N/A
	Tone/rigidity	✓	X	X	To observe arm tone, UTA can sway standing patient at the hip.
	Test of subtle paresis	✓	✓	✓	Pronator drift, forearm rolling test, and velocity and cadence of movement
	Muscle strength	✓	Limited	X	Can still note symmetry, velocity, and functional tests unassisted
**Sensory**					
	Light touch	✓	✓	Limited	Unassisted patients can simultaneously touch both upper or lower extremities.
	Pain/temperature	✓	X	X	N/A
	Vibration/proprioception	✓	X	X	N/A
	Spinal sensory levels	✓	✓	X	N/A
Reflexes		✓	X	X	DTRs^f^, Plantar response, Hoffman’s, abdominal reflexes, primitive reflexes, clonus
**Cerebellar**					
	Appendicular	✓	✓	✓	Includes heel to shin, finger to nose, rapidly alternating movement
	Truncal	✓	X	X	N/A
Gait		✓	X	X	Per physician discretion, standing and ambulation may be assessed unassisted or with untrained assistant, depending on perceived fall risk. TA may assess heel walking, toe walking, tandem gait, and Romberg safely.

^a^TA: tele-exam with trained assistant.

^b^UTA: tele-exam with untrained assistant.

^c^UA: tele-exam unassisted.

^d^N/A: not applicable.

^e^SCM: sternocleidomastoid.

^f^DTR: deep tendon reflex.

## Conclusions

Adequate telemedical history taking and physical examination performance depend on the patient, availability of an assistant, and the assistant’s level of training. A hands-on educational curriculum to train assistants has the potential to narrow the gap between in-person and telemedicine examinations. This could substantially increase access to higher-level expert evaluations across the United States and internationally. More research regarding training, costs, implementation, and outcome measures for such assistants is warranted. As telemedicine continues to grow rapidly, the field must remain proactive, flexible, and nimble in using all resources to improve quality, access, and value to our patients.

## Appendix

Multimedia Appendix 1 Detailed breakdown of the neurological examination elements laid out in Table 1.

## Abbreviations

APP: advanced practice providers

## References

[1] Hatcher-Martin JM, Adams JL, Anderson ER, Bove R, Burrus TM, Chehrenama M, Dolan O'Brien M, Eliashiv DS, Erten-Lyons D, Giesser BS, Moo LR, Narayanaswami P, Rossi MA, Soni M, Tariq N, Tsao JW, Vargas BB, Vota SA, Wessels SR, Planalp H, Govindarajan R (2020). Telemedicine in neurology: Telemedicine Work Group of the American Academy of Neurology update. Neurology.

[2] Weinstein RS, Krupinski EA, Doarn CR (2018). Clinical examination component of telemedicine, telehealth, mHealth, and connected health medical practices. Med Clin North Am.

[3] Cohen B, Hyman S, Rosenberg L, Larson E (2012). Frequency of patient contact with health care personnel and visitors: implications for infection prevention. Jt Comm J Qual Patient Saf.

[4] (2016). Telehealth Facilitator Certificate. Thomas Jefferson University.

[5] CTCP: Certified Telemedicine Clinical Presenter. Southside Telelehealth Training Academy and Resource Center.

[6] Li Y, Li N, Han Q, He S, Bae RS, Liu Z, Lv Y, Shi B (2014). Performance of physical examination skills in medical students during diagnostic medicine course in a University Hospital of Northwest China. PLoS One.

[7] Craig JJ, McConville JP, Patterson VH, Wootton R (1999). Neurological examination is possible using telemedicine. J Telemed Telecare.

[8] Al Hussona M, Maher M, Chan D, Micieli JA, Jain JD, Khosravani H, Izenberg A, Kassardjian CD, Mitchell SB (2020). The virtual neurologic exam: instructional videos and guidance for the COVID-19 era. Can J Neurol Sci.

[9] Telemedicine and COVID-19 implementation guide. American Academy of Neurology.

[10] (2020). Quick Safety: The optimal use of telehealth to deliver safe patient care. The Joint Commission.

[11] Telehealth Implementation Playbook. American Medical Association.

[12] Thaller M, Hughes T (2014). Inter-rater agreement of observable and elicitable neurological signs. Clin Med (Lond).

[13] Awadallah M, Janssen F, Körber B, Breuer L, Scibor M, Handschu R (2018). Telemedicine in general neurology: interrater reliability of clinical neurological examination via audio-visual telemedicine. Eur Neurol.

[14] Sarangi S, Wynn R (2015). Editorial: telemedicine/e-health as mediated communication. Commun Med.

[15] Lindauer A, Seelye A, Lyons B, Dodge HH, Mattek N, Mincks K, Kaye J, Erten-Lyons D (2017). Dementia care comes home: patient and caregiver assessment via telemedicine. Gerontologist.

[16] Schlaak H (2018). Professional competencies for e-helpers: a telepractice resource. Theses Dissertations--Commun Sci Disord.

