# Supplementary material for: Certified Examination Assistants in the Age of Telemedicine: A Blueprint Through Neurology
Source: JMIR Med Educ. 2021 Oct 6;7(4):e28335. doi: 10.2196/28335 (PMC8529478; doi:10.2196/28335)
Supplement: Multimedia Appendix 1 [file mededu_v7i4e28335_app1.docx]

## Appendix 1

There are few restrictions on mental status assessments. With cognitively intact individuals or those with mild dementia, remote evaluation may include (but is not limited to) the use of pen and paper for writing. More advanced telemedicine software can enable patients to engage directly with the unit (transmitting handwriting, drawings, or other notes) in real-time that the neurologist can view on his or her screen. It is important to note that high kappa scores (0.69–0.93) were reported for generalized cognitive testing with slight adjustments tailored to telemedicine patients [15]. Additional cognitive testing would need to be validated in terms of unassisted examinations. Higher cortical function such as gnosis would not be assessed unassisted. However, a detailed cognitive examination by a skilled neurologist with an assistant would be sufficient for most cognitive assessments. Cranial nerve assessment is somewhat limited without assistance but not greatly contingent upon assistant skill. Performing swallowing assessment on the vast majority of neurological patients is low risk, even without a trained assistant. That being said, swallowing assessment would typically involve tiny sips of water first which confers little to no risk, even with dysphagia. A motor examination would begin similar to an IPE with the observation of bulk and atrophy, movement fluidity, coordination, and velocity. Motor strength grading can be performed with a certified assistant like an in-person evaluation using the Medical Research Council Manual Muscle Testing scale, a 0–5-point motor strength scale. As with IPEs, the biggest limitation during a telemedicine evaluation is in the 4 of 5 strength range where there is some weakness, but still antigravity strength with different examiners using different ways to note differences within this range. Without an assistant, testing with pronator drift, speed, and symmetry of movements, and functional maneuvers like chair stand testing and timed up and go testing can serve as a proxy. Sensory examination at a gross level can be self-performed by a patient or untrained assistant checking light touch, but a trained assistant is necessary to conduct a detailed sensory examination using multiple sensory modalities. Reflex examination, including evaluation of deep tendon reflexes, pathological reflexes, and assessment for clonus, would require a trained assistant. Therefore, without a trained assistant, a detailed evaluation of these two subsections would be limited. Results without a trained assistant may be unclear and of little value, even if recorded. A cerebellar examination can be conducted as usual with minimal assistance, given a relatively cognitively intact patient. Although not requiring much skill on the examiner side, gait testing confers a fall risk and should only be assessed in the presence of an assistant in any patient with ambulatory or cognitive dysfunction.
